# Phosphoglucoisomerase Is an Important Regulatory Enzyme in Partitioning Carbon out of the Calvin-Benson Cycle

**DOI:** 10.3389/fpls.2020.580726

**Published:** 2020-12-10

**Authors:** Alyssa L. Preiser, Aparajita Banerjee, Sean E. Weise, Luciana Renna, Federica Brandizzi, Thomas D. Sharkey

**Affiliations:** ^1^MSU-DOE Plant Research Laboratory, Michigan State University, East Lansing, MI, United States; ^2^Department of Biochemistry and Molecular Biology, Michigan State University, East Lansing, MI, United States; ^3^Department of Plant Biology, Michigan State University, East Lansing, MI, United States; ^4^Plant Resilience Institute, Michigan State University, East Lansing, MI, United States

**Keywords:** phosphoglucoisomerase, starch, Calvin-Benson cycle, carbon partitioning, erythrose 4-phosphate

## Abstract

Phosphoglucoisomerase (PGI) isomerizes fructose 6-phosphate (F6P) and glucose 6-phosphate (G6P) in starch and sucrose biosynthesis. Both plastidic and cytosolic isoforms are found in plant leaves. Using recombinant enzymes and isolated chloroplasts, we have characterized the plastidic and cytosolic isoforms of PGI. We have found that the *Arabidopsis* plastidic PGI *K*_*m*_ for G6P is three-fold greater compared to that for F6P and that erythrose 4-phosphate is a key regulator of PGI activity. Additionally, the *K*_*m*_ of spinach plastidic PGI can be dynamically regulated in the dark compared to the light and increases by 200% in the dark. We also found that targeting *Arabidopsis* cytosolic PGI into plastids of *Nicotiana tabacum* disrupts starch accumulation and degradation. Our results, in combination with the observation that plastidic PGI is not in equilibrium, indicates that PGI is an important regulatory enzyme that restricts flow and acts as a one-way valve preventing backflow of G6P into the Calvin-Benson cycle. We propose the PGI may be manipulated to improve flow of carbon to desired targets of biotechnology.

## Introduction

Partitioning of carbon out of central metabolism toward desired end-products is a key feature of biotechnological efforts in plants. This may be directed toward increased yields of starch, carotenoids, novel compounds, or other products (see Botella-Pavía and Rodríguez-Conceptión, 2006; Ausich, 2009; Santelia and Zeeman, 2011; and Abiri et al., 2016 for examples). In normal plant physiology, plants partition carbon out of the Calvin-Benson cycle to starch synthesis. This must be a regulated process as carbon must be carefully partitioned out of the Calvin-Benson cycle in order to not deplete metabolite pools needed for continuation of the Calvin-Benson cycle while still accumulating adequate amounts of starch to survive the night (Sulpice et al., 2014). Study of the regulation of carbon partitioning to starch synthesis can provide insight how plastid metabolism is regulated to control how much carbon stays in the Calvin-Benson cycle. This is essential for future biotechnological applications in order to maintain photosynthetic metabolite pools to support carbon assimilation and to redirect carbon to desired end products.

While starch synthesis has been well-studied for decades, our knowledge of the regulation of the entire pathway remains incomplete. The primary pathway for carbon conversion to starch is by action of the plastid phosphoglucoisomerase (PGI) converting fructose 6-phosphate (F6P) of the Calvin-Benson cycle to glucose 6-phosphate (G6P), forming a branch point out of the Calvin-Benson cycle and initiating starch synthesis. Phosphoglucomutase then converts G6P to glucose 1-phosphate and ADP glucose pyrophosphorylase uses the glucose 1-phosphate to make ADPglucose, the substrate for starch synthases. The regulation of PGI, the enzyme that partitions carbon out of the Calvin-Benson cycle, is not clearly understood (Bahaji et al., 2015). This reaction is reversible but is displaced from equilibrium (Schnarrenberger and Oeser, 1974; Gerhardt et al., 1987; Sharkey and Vassey, 1989; Backhausen et al., 1997; Szecowka et al., 2013) indicating that it is kinetically limited. What is more, a similar degree of disequilibrium is found at different rates of photosynthesis indicating that it is regulated (Schleucher et al., 1999). Additionally, it has been shown that plastidic PGI has a higher *K*_*m*_ for G6P than for F6P (Schnarrenberger and Oeser, 1974). The cytosolic isoform, which is involved in sucrose synthesis, has neither of these characteristics. It is unknown why the plastidic PGI is displaced from equilibrium and appears to be regulated, despite catalyzing an easily reversible reaction. We explore the regulation of PGI using recombinant enzymes and reexamine some previously held assumptions about PGI regulation. We confirm erythrose 4-phosphate (E4P) as a key regulator, show that the *K*_*m*_ of PGI is dynamically regulated in the dark and light, and demonstrate that manipulating the regulation of PGI can change the accumulation of starch.

## Materials and Methods

### Overexpression and Purification of Recombinant Enzymes

N-terminal His-tagged *Arabidopsis thaliana* plastidic (with targeting peptide removed) and cytosolic PGI cDNA sequences were commercially synthesized by GenScript^1^. Both constructs were placed in separate pET11a bacterial expression vectors (Millipore-Sigma, Burlington, MA, United States) and were overexpressed in *Escherichia coli* strain BL21. Cells were grown at 37°C to an OD_600_ of 0.6 to 1.0 and induced with 0.5 mM isopropyl β-D-1 thiogalactopyranoside (IPTG) at room temperature overnight. Cells were centrifuged and resuspended in lysis buffer (5 mL lysis buffer/g of pellet; 50 mM sodium phosphate, pH 8.0, 300 mM NaCl) containing 1 mg mL^–1^ lysozyme, 1 μg mL^–1^ of DNAseI, and 1x protease inhibitor cocktail (Sigma Aldrich)^2^. Cells were then lysed by sonication (Branson Sonifier 250)^3^. The sonicator was set at 50% duty cycle and an output level of 1. The cells were sonicated using five steps where each step consisted of a 15 s pulse followed by 15 s on ice. The lysate was centrifuged and supernatant collected. Ni-NTA resin (0.25 volume of lysate; Qiagen)^4^ was added to the crude lysate with gentle stirring for 1 h. The mixture was loaded into a column and allowed to settle, then washed with wash buffer (50 mM sodium phosphate, pH 8.0, 300 mM NaCl, 10 mM imidazole) until the OD_280_ of the effluent was less than 0.05. Protein was eluted with six volumes of elution buffer (50 mM sodium phosphate pH 8.0, 300 mM NaCl, 250 mM imidazole) containing 1x protease inhibitor cocktail [Sigma Aldrich (see text footnote 2)]. The Ni-NTA column purification was performed in a cold room at 4°C. For all purified proteins, protein concentration was determined using a Pierce 660 nm protein assay reagent kit (Thermo Fisher Scientific)^5^ using a bovine serum albumin standard. Fractions containing >95% of the protein of interest were combined and concentrated using Amicon Ultra 0.5 ml centrifugal filters (molecular weight cut off of 3 kDa). Glycerol was added to the concentrated protein to obtain a final protein solution with 15% glycerol. The glycerol stock of the proteins was aliquoted into small volumes, frozen in liquid nitrogen, and stored at –80°C. Final preparations of purified protein were run on a 12% SDS-polyacrylamide gel and stained with Coomassie Blue to check the purity of the enzymes and concentration was determined as described above. Molecular weights were estimated from the protein construct using Vector NTI [Thermo Fisher Scientific (see text footnote 5)].

### Coupled Spectrophotometric Assay for PGI (F6P to G6P Reaction)

The activity of the purified plastidic and cytosolic PGI was studied using coupled spectrophotometric assays. Concentrations of G6P and F6P were measured spectrophotometrically using NADPH-linked assays (Lowry and Passonneau, 1972). All assays were validated by demonstrating linear product formation, proportional to the time of the assay and amount of enzyme added. All coupling enzymes (glucose-6-phosphate dehydrogenase (G6PDH) for the F6P to G6P direction and phosphofructokinase for the G6P to F6P direction) were added in excess so that no change in product formation was seen when varying the coupling enzyme. PGI assays were done in 50 mM bicine buffer pH 7.8, containing 4.8 mM DTT, 0.6 mM NADP^+^, 2 U glucose-6-phosphate dehydrogenase (G6PDH) (from *Leuconostoc mesenteroides* Sigma-Aldrich catalog number G8529), varying concentrations of F6P as indicated below, and 1.31 ng plastidic or cytosolic PGI. The reaction was:

$$\begin{array}{c} \text{F}6\text{P}⇄\text{G}6\text{P}+{\text{NADP}}^{+}\to 6-\text{phosphogluconate}\\ +\text{NADPH}+{\text{H}}^{+} \end{array}$$

The concentrations used to study the *K*_m_ of PGI for F6P were 0-4.8 mM. Under these conditions, less than 5% of the non-limiting substrate was consumed over the duration of the assay. The assay mixtures were prepared by adding all the components except the enzyme. Activity was recorded with a dual wavelength filter photometer (Sigma ZFP2) as the increase in absorbance at 334 nm minus absorbance at 405 nm caused by NADP^+^ reduction to NADPH using an extinction coefficient of 6190 M^–1^ cm^–1^. These wavelengths were used because they correspond to emission wavelengths of the lamp used in the filter photometer.

### Mass Spectrometry Assay for PGI (G6P to F6P Reaction)

The activity of the purified plastidic and cytosolic PGI in the G6P to F6P direction was studied using a coupled mass spectrometer assay. The assay mixture contained 50 mM Tris pH 7.8, 2.5 mM MgCl_2_, 1 mM ATP, 5 mM DTT, 0.15 U phosphofructokinase (from *Bacillus stearothermophilus* from Sigma Aldrich catalog number F0137), varying concentrations of G6P from 0 to 3.6 mM, and 1.6 ng of plastidic or cytosolic PGI.

The reaction was:

G6P⇄F6P→FBP

The assay mixtures were prepared by adding all the components except the enzyme. The reaction was initiated upon addition of the enzyme. After 5 min, the reaction was quenched with four volumes of 100% ice-cold methanol. The concentration of fructose-1,6-bisphosphate (FBP) produced was shown to be linear for up to 10 min. Five nmol of D-[UL-^13^C_6_] FBP was added as an internal standard for quantification, and the sample was heated for 5 min at 95°C. Six volumes of 10 mM tributylamine, pH 5.0, was added and the sample was filtered through a Mini-UniPrep 0.2 μm Syringeless Filter Device (GE Healthcare Life Sciences, Whatman). LC/MS-MS was carried out on a Waters Quattro Premier XE system and was operated in electrospray negative ion mode with multiple reaction monitoring (Table 1). The capillary voltage was 2.75 kV; the cone voltage, 50 V; the extractor voltage, 5 V. The source temperature was 120°C and the desolvation temperature was 350°C. Gas flow for the desolvation and cone was set to 800 and 50 L h^–1^, respectively. MassLynx software and the Acquity UPLC Console were used to control the instrument. Samples were passed through an Acquity UPLC BEH Column (Waters) with a multi-step gradient with eluent A (10 mM tributylamine with 5% methanol, adjusted to pH 6 with 500 mM acetic acid) and eluent B (methanol): 0–1 min, 95–85% A; 1–3 min, 85–65% A; 3–3.5 min, 65–40% A; 3.5–4 min, 40–0% A; 4–8.50 min, 0% A; 8.5–10 min, 100% A. The flow rate was 0.3 mL min^–1^. FBP peaks were integrated using QuanLynx software and the concentration of the metabolites was quantified by comparing the peak response to an external calibration curve.

**TABLE 1:** Parameters used for detection of fructose bisphosphate (FBP) and the internal standard with LC/MS/MS.

Metabolite	Cone (V)	Collision (V)	+0 Parent (m/z)	Daughter (m/z)
FBP	26	18	339	97
D-[UL-^13^C_6_] FBP	26	18	345	97

*Parameters were optimized using 10 μM standards before analyzing samples.*

### Kinetic Characterization

Enzymes were assayed at varying concentrations of substrate as described above. The *K*_*m*_ values for plastidic and cytosolic PGI were determined by fitting the data with non-linear regression using the Hill function in OriginPro 8.0 (OriginLab Corporation).

### Inhibition Studies

Different metabolites of the Calvin-Benson cycle were tested for their effect on PGI activity. All the metabolites were purchased from Sigma Aldrich [Sigma Aldrich (see text footnote 2)]. In metabolite screening assays, metabolites were assayed at a 1:1 molar ratio with the substrate. To determine the *K*_*i*_ of PGI for different metabolites, the assay was carried out in presence of various concentrations of F6P or G6P and the inhibitory metabolite. Assay mixtures were prepared as described above. In inhibition assays, 0–0.98 mM F6P or 0–1.5 mM G6P was used. The concentration range used to study the *K*_*i*_ of PGI for E4P was 0–0.05 mM and that for 6PG was 0–1.5 mM. The mechanism of inhibition was determined from Hanes–Woolf plots. The *K*_*i*_ was determined from the non-linear least squares fitting of the activity vs. concentration plot using Solver in Excel using the standard equation for competitive inhibition as described below:

(1) $$v=\frac{V_{\max}^{*}S}{K_{m}\left(1+\frac{I}{K_{i}}\right)+S}$$

where *V*_max_ is the maximum velocity, *S* is the substrate concentration, *K*_*m*_ is the Michaelis constant, and *K*_i_ is the inhibition constant. For non-competitive inhibition, the equation below was used.

(2) $$v=\frac{V_{\max}^{*}S/\left(1+\frac{I}{K_{i}}\right)}{\left(K_{m}\left(1+\frac{I}{K_{i}}\right)/\left(1+\frac{I}{K_{i}}\right)+S\right)}$$

### Plant Material

Fresh *Spinacia oleracea* (So) was purchased at a local market for use that day. Spinach was either dark or light treated for 1.5 h before beginning isolation and petioles were kept in water to prevent wilting.

*Arabidopsis thaliana* (At) Col-0 was grown in SureMix soil (Michigan Grower Products, Inc., Galesburg, MI, United States) in a growth chamber at a 12 h light at 120 μmol m^–2^ s^–1^, 23°C and 12 h dark at 21°C. Plants were harvested either midday for light samples or midnight for dark samples.

*Nicotiana tabacum* seeds were planted in SureMix soil. The plants were grown in the greenhouse, starting in October 2019 with an average daytime temperature of 27°C and nighttime temperature of 20°C. Plants were fertilized twice per week with commercially available Peters 20-20-20 fertilizer (ICL Specialty Fertilizers)^6^ at 100 ppm. Experiments were done when plants were 5–12 weeks old on the fifth to seventh fully expanded leaves.

### Chloroplast Isolation

Chloroplasts were isolated using a Percoll gradient (Weise et al., 2004). Leaves were placed in a chilled blender with grinding buffer (330 mM mannitol, 50 mM Hepes, pH 7.6, 5 mM MgCl_2_, 1 mM MnCl_2_, 1 mM EDTA, 5 mM ascorbic acid, 0.25% BSA), blended, and then filtered through four layers of cheese cloth. Filtered liquid was centrifuged, and the pellet was resuspended in resuspension buffer (330 mM mannitol, 50 mM Hepes, pH 7.6, 5 mM MgCl_2_, 1 mM MnCl_2_, 1 mM EDTA, 0.25% BSA). The resuspended pellet was layered on top of a 20–80% Percoll gradient which was centrifuged at 1,200 × *g* for 7 min. The bottom band in the gradient containing the intact chloroplasts was collected. One volume of resuspension buffer was added to the collected chloroplasts and centrifuged at 1,200 × *g* for 2 min. The pellet was resuspended in 50 μL of water and vortexed to lyse the chloroplasts. One volume of 2x buffer (100 mM Hepes, pH 7.6, 10 mM MgCl_2_, 2 mM MnCl_2_, 2 mM EDTA, 2 mM EGTA, 60% glycerol, 0.2% Triton X-100, 0.2% PVPP) was added. Samples were stored at –80°C until used for further analysis. Chlorophyll was quantified by lysing 50 μL of purified chloroplasts by sonication and adding supernatant to 1 mL of 95% ethanol. OD_654_ was used to calculate the chlorophyll concentration (Wintermans and DeMots, 1965):

(3) $$\text{mg}\text{Chl}=OD^{*}0.0398^{*}0.050\mu \text{L}$$

Assays that used isolated chloroplasts were normalized by amount of chlorophyll added to the assay mixture.

### Transient Expression of PGI in *N. tabacum*

A fusion gene was generated using the transit peptide of the *Arabidopsis* chloroplast *PGI1* (*At4g24620*). This was determined by ChloroP^7^ to be the first 144 bp starting at the ATG codon. The transit peptide was placed in front of the *Arabidopsis* cytosol *PGI2* (At5g42740) cDNA with the ATG from the *PGI2* sequence omitted, for a total length of 1824 bp. A second fusion gene was made by placing a YFP gene sequence at the end of the gene directly before the TGA stop codon. A third fusion gene was made by placing the YFP gene sequence at the N-terminus between the transit peptide and the *PGI2* cDNA sequence. These constructs were synthesized by Bio Basic Inc (Markham, ON, Canada) and placed in the pUC57 plasmid vector. The construct was then transferred to the pEAQ-HT-DEST1 destination vector containing the P19 suppressor of silencing (Sainsbury et al., 2009). The pEAQ-HT-DEST1 vector constructs were transformed into *Agrobacterium* strain GV3101 by electroporation. All constructs and transformed *E. coli* and *Agrobacterium* were confirmed with PCR.

*Agrobacterium* containing the desired construct was grown in LB media in a 5 mL culture tube at 28°C overnight. The next day cells were pelleted by centrifugation at 7,000 × *g* for 5 min at 20°C and washed twice with infiltration buffer (2 mM trisodium phosphate, 50 mM MES, 25 mM glucose, 200 mM acetosyringone). The OD_600_ was measured and used to calculate the necessary volume of *Agrobacterium* to dilute to an OD_600_ of 0.05, 0.025, or 0.01 for initial controls and 0.025 for all subsequent experiments. *N. tabacum* leaves were gently infiltrated using a 1 mL syringe without a needle according to Batoko et al. (2000). All experiments, except for initial controls, were done at 2 days post-infiltration.

### Localization of Retargeted PGI

Protein transient expression was performed using 4-week-old *N. tabacum* plants and *Agrobacterium tumefaciens* (strain GV3101) with an OD_600_ of 0.05. Both N-terminal and C-terminal YFP fusion constructs were used.

Confocal images were acquired using an inverted laser scanning confocal microscope, Nikon A1RSi, on tobacco leaf epidermal cells, 2 days post-infiltration. Images were acquired using a 60X oil λS DIC N2 objective. YFP was excited by the 514 nm line of an argon ion laser and emission collected at 530–560 nm. Chlorophyll autofluorescence was excited with the 647 nm line and emission was collected at 680–750 nm (Mehrshahi et al., 2013).

### Starch Time Course

*Nicotiana tabacum* leaves were infiltrated as described above. Starting 48 h post-infiltration, leaf punches were collected at 4:40 PM (+10:40 h after lights on), 10:00 PM (lights off), 12:40 PM (+ 2:40 h after lights off), 3:20 AM (+5:20 h after lights off), 6:00 AM (lights on), and 11:20 AM (+5:20 h after lights on). These times corresponded with lights on, lights off, and two points evenly spaced in between each change in light conditions. Samples were collected in 2 mL pre-weighed microcentrifuge tubes, frozen immediately in liquid nitrogen, and stored at −80°C. Fresh weight was determined before any further analysis.

Frozen plant material was ground using a ball mill (Retsch)^8^ and was suspended in ice-cold 3.5% perchloric acid solution (50% w/v of plant tissue), homogenized, and incubated on ice for 5 min. Samples were then centrifuged at 28,000 × *g* for 10 min at 4°C. The pellet was washed twice with both 80% ethanol and deionized water and then dried in a Savant AES 1010 SpeedVac [Thermo Fisher Scientific (see text footnote 5)] for 15 min to remove any remaining ethanol. The dried pellet was resuspended in 500 μL of 0.2 M KOH and incubated at 95°C for 30 min to gelatinize the starch. Acetic acid was added to a final concentration of approximately 150 mM to bring the solution to a pH of 5. Fifty U of α-amylase and 0.2 U of amyloglucosidase were added to the sample. The starch solution was incubated for 2 days at room temperature on a shaker to convert the starch to glucose. Two hundred μL of an assay mixture of 110 mM HEPES, 500 nmol NADP^+^, 500 nmol ATP, and 0.4 U G6PDH was added to wells in an assay plate. Twenty μL of the digested starch sample was added to each well and was measured at 340 nm on a FilterMax F5 Plate Reader (Molecular Devices)^9^ until a stable baseline was obtained. A starting OD_340_ was measured. One U of hexokinase was added to each well, quickly shaken, and monitored at 340 nm until the reaction was completed. An endpoint OD_340_ was measured and starch (glucose equivalents) was determined using the ΔOD.

Significance of linearity for starch degradation at night was determined by linear regression statistics in OriginPro 8.0 (OriginLab Corporation).

## Results

### Plastidic and Cytosolic PGI Have Different Kinetic Properties

Purified *Arabidopsis* plastid and cytosolic PGI, produced as described above, had specific activities of 787 μmol mg^–1^ protein min^–1^ for plastidic PGI and 1522 μmol mg^–1^ protein min^–1^ for cytosolic PGI. Table 2 shows the *K*_*m*_ (for both F6P and G6P) of plastidic and cytosolic PGI (Supplementary Figure 2). For plastidic AtPGI, the *K*_m_ for G6P was ∼2.9-fold higher than that for F6P. The *K*_*m*_’s for F6P and G6P of the cytosolic enzyme were the same. We did not calculate the *V*_*max*_ value of G6P and F6P for both isoforms since initial results did not conform to the Haldane relation (Alberty, 1953):

(4) $$K=\frac{k_{\mathrm{cat}\mathrm{F6P}\to \mathrm{G6P}}}{k_{catF6P\to G6P}}*\frac{k_{mG6P\to F6P}}{k_{mG6P\to F6P}}$$

**TABLE 2:** Kinetic constants and inhibition constants for plastidic and cytosolic AtPGI as determined by NADPH-linked spectrophotometric assays and LC-MS/MS assays.

	F6P G6P		G6P F6P	
	Plastidic PGI	Cytosolic PGI	Plastidic PGI	Cytosolic PGI
*K*_m_ (μM)	73 ± 80	203 ± 12	164 ± 43	158 ± 85
E4P *K*_i_ (μM)	2.3	1.5	6.0	3.7
6PG *K*_i_ (μM)	31	106	245	149

*K_m_ and k_cat_ were determined by fitting the Michaelis-Menten equation. Data points used in model fitting were different preparations (n = 3). For inhibition constants, each number was determined from the fitted curves as described in the methods. Errors shown are SD, (n = 3). Sum of least-squares for inhibition parameters were determined using Solver in Excel.*

Therefore, we concluded that the measured *V*_max_ was not reliable, either due to differences in methodology for measuring G6P or F6P kinetics or storage in the freezer. Using the determined *K*_*eq*_ of 3.70 (Dyson and Noltmann, 1968), we can calculate that the ratio of *k*_*cat F*6*P*→*G*6*P*_/*k*_*cat G*6*P*→*F*6*P*_ is 1.65 for the plastidic isoform and 4.75 for the cytosolic isoform. DTT did not significantly influence the specific activity of plastidic or cytosolic AtPGI (Supplementary Figure 3).

### Regulation of PGI Activity

**FIGURE 1:**
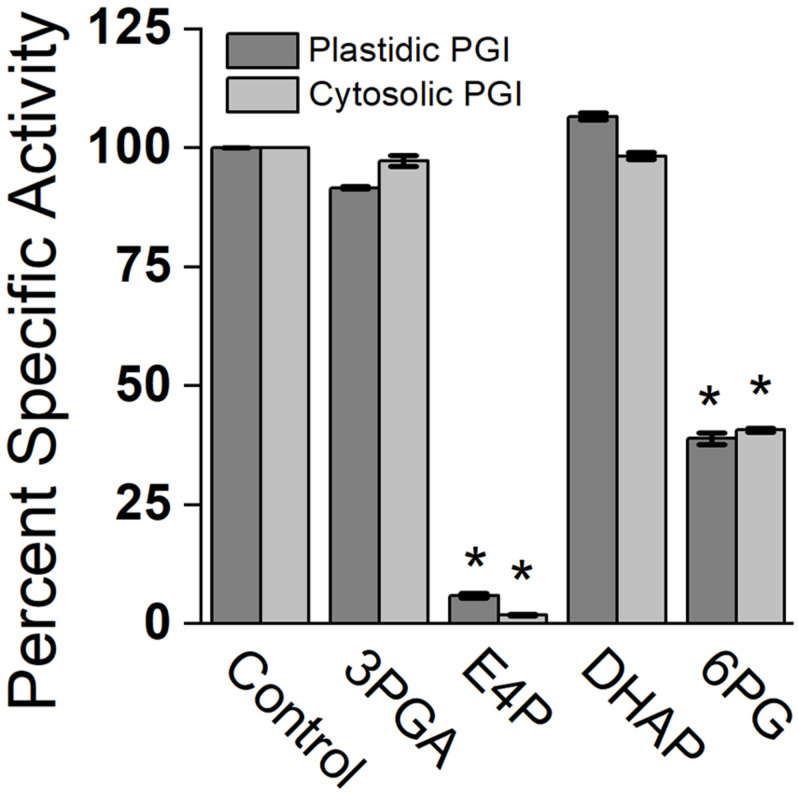
Comparison of specific activity of plastidic and cytosolic AtPGI with various metabolites. Each bar represents mean and error bars represent SD, (*n* = 3). All metabolites were screened at 1:1 F6P substrate to metabolite. Data with an asterisk (*) are significantly different from the control as determined by Student’s *t*-test (*P* < 0.05) (two tailed and assuming unequal variance). 3PGA, 3-phosphoglyceric acid; E4P, erythrose 4-phosphate; DHAP, dihydroxyacetone phosphate; 6PG, 6 phosphogluconate.

**FIGURE 2:**
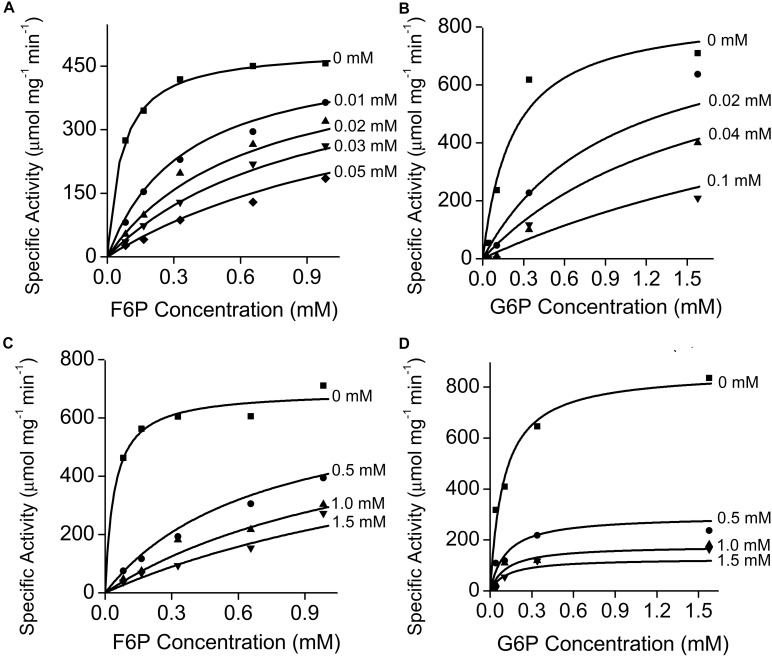
Effect of erythrose 4-phosphate (E4P) and 6-phosphogluconate (6PG) on plastidic AtPGI. We measured the effect of E4P **(A,B)** and 6PG **(C,D)** on AtPGI. Different symbols represent different concentrations of inhibitor. PGI was more inhibited by E4P than by 6PG. Lines represent data fit to Eq. 2. F6P, fructose 6-phosphate; G6P, glucose 6-phosphate.

Effects of different metabolites on the activity of PGI was similar for both plastidic and cytosolic AtPGI. Inhibition with erythrose 4-phosphate (E4P), 3-phosphoglyceric acid (PGA), dihydroxyacetone phosphate (DHAP), and 6-phosphogluconate (6PG) were screened (Figure 1). Only E4P and 6PG showed significant inhibition of PGI activity. Figure 2 shows the activity of plastidic AtPGI over a range of 6PG and E4P concentrations. Activity of cytosolic AtPGI was analyzed in a similar manner as shown for plastidic AtPGI. The calculated *K*_*i*_ values of E4P and 6PG are shown in Table 2. The *K*_*i*_ values for 6PG were between 31 and 203 μM, depending on the isoform and substrate. E4P was shown to be more inhibitory with *K*_*i*_’s between 1.5 and 6 μM. Based on the Hanes-Woolf plots (Supplementary Figure 4), E4P was shown to be competitive, except above 0.04 mM, with G6P. 6PG was identified as competitive with F6P, except above 1.0 mM, and non-competitive with G6P.

**FIGURE 3:**
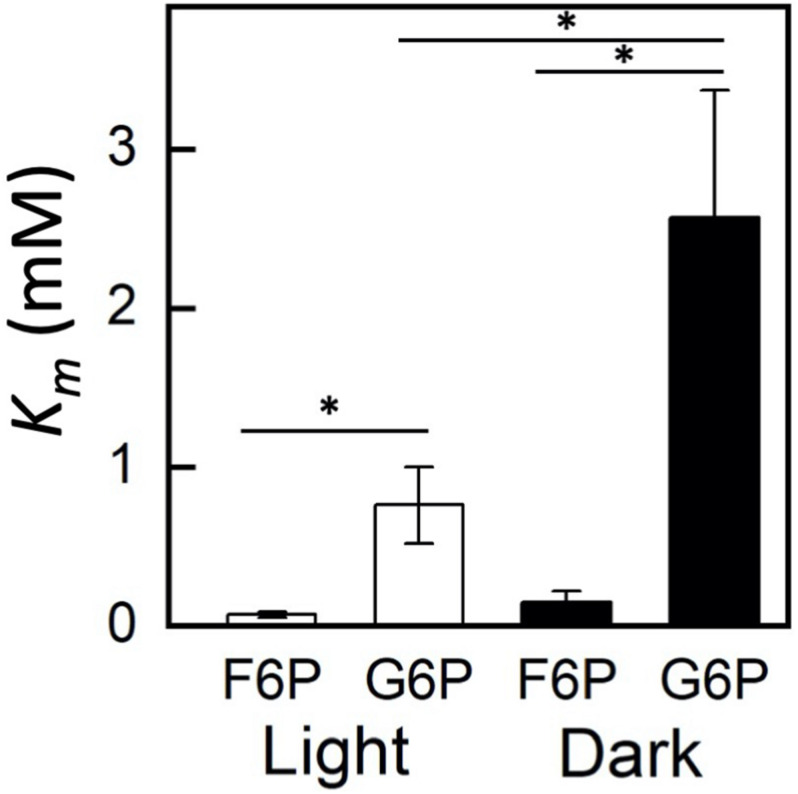
Comparison of fructose 6-phosphate (F6P) and glucose 6-phosphate (G6P) *K*_m_ in plastidic phosphoglucoisomerase in dark and light-treated isolated spinach chloroplasts. Each bar represents mean and error bars represent SE, (*n* = 3 independent isolations of chloroplasts). The *K*_m_ for G6P increased in dark treated compared to light treated isolated chloroplasts. Bars with an asterisk (*) are significantly different from corresponding light treated samples as determined by Student’s *t*-test (*P* < 0.05) (two tailed and assuming unequal variance).

Plastidic SoPGI activity from chloroplasts from dark-treated spinach leaves had a higher *K*_*m*_ for G6P compared to light-treated chloroplasts (Figure 3). The *K*_*m*_ of SoPGI for F6P did not change in the light or dark.

### Retargeted Cytosolic AtPGI Localized in the Chloroplast

**FIGURE 4:**
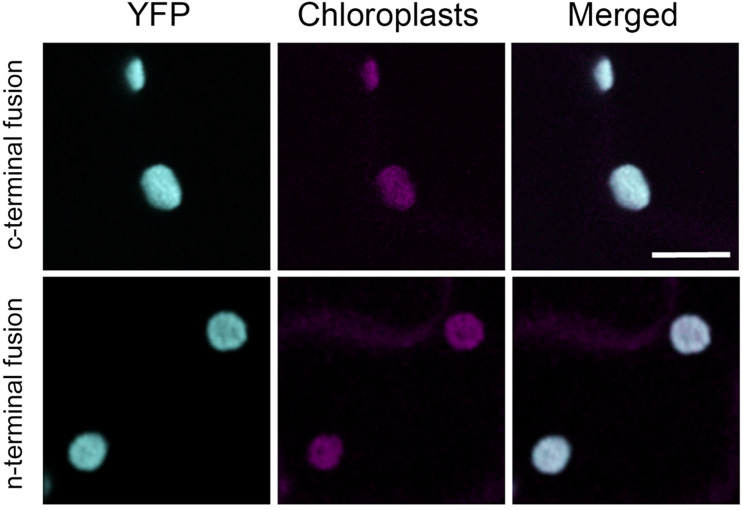
Localization of modified plastid phosphoglucoisomerase (cPGI) to the chloroplast. Confocal images of *Nicotiana tabacum* epidermal cell transiently expressing N- or C-terminal tagged mislocalized PGI. Both fluorescent fusions localize at the chloroplasts (cyan) as the co-localization (merge channel) shows with the chloroplast autofluorescence (magenta). Scale bar 10 μm.

We transiently expressed retargeted cytosolic PGI. To confirm the intracellular location, we added YFP proteins on both the N-terminus and C-terminus. Tobacco leaf epidermes were observed by confocal microscopy. YFP fluorescence was seen in the chloroplast for both the N-terminal and C-terminal constructs, confirming that our construct of cytosolic AtPGI targeted to the plastids was in the chloroplast (Figure 4).

After infiltrating *N. tabacum* with *Agrobacterium* transformed with the modified AtPGI construct without the YFP addition, we collected samples every 24 h for 3 days. Expression of the construct was highest on the second day after infiltration as determined by quantitative PCR (Supplementary Figure 5A). We also found that an *Agrobacterium* density of 0.025 OD_600_ resulted in the highest expression of the construct (Supplementary Figure 5). Photosynthetic assimilation was ∼50% of pre-infiltration values (Supplementary Figure 5B). After 2 days both expression and photosynthesis declined. Based on this, we used 2 days post-infiltration as the time point for all future experiments.

### Time Course of Starch Accumulation in *N. tabacum* Transiently Expressing Retargeted AtPGI

**FIGURE 5:**
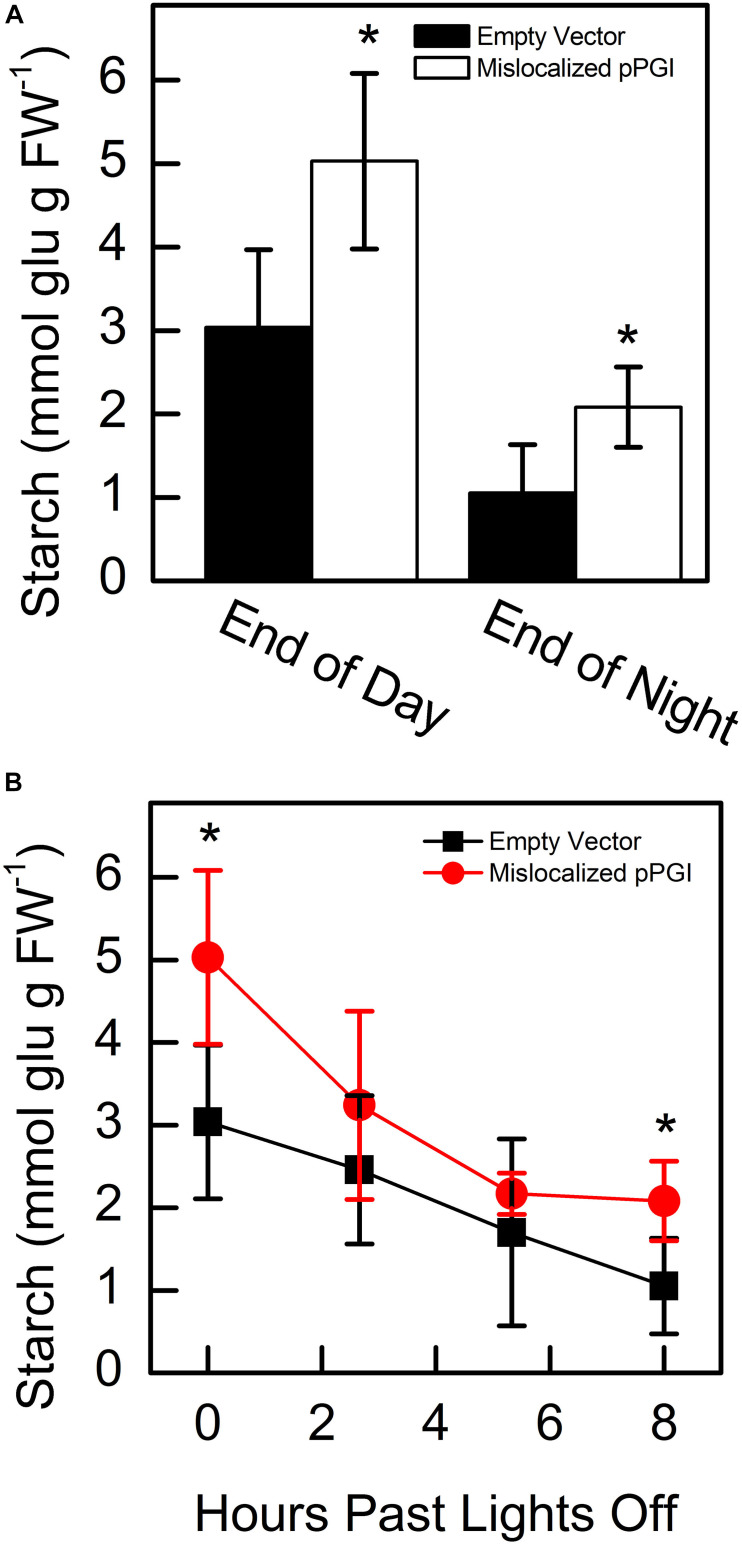
Starch amounts at end of day and end of night **(A)** and degradation of starch **(B)** in *Nicotiana tabacum* expressing mislocalized plastid phosphoglucoisomerase (pPGI). Starch in plants with mislocalized pPGI was significantly higher than empty vector control plants. Starch breakdown in empty vector plants was linear through the night, however, starch breakdown was non-linear in mislocalized pPGI plants. Each bar or data point represents mean and error bars represent SE, (*n* = 5 biological replicates). Bars or data points with an asterisk (*) are significantly different from corresponding empty vector samples as determined by Student’s *t*-test (*P* < 0.05) (two tailed and assuming unequal variance). Significance for linearity was determined by Prob(F) of the linear regressions.

Starting 48 h after infiltration, we measured starch content in *N. tabacum* that transiently expressed either the mislocalized PGI construct or an empty vector. We found that at the end of day and end of night, PGI plants had significantly more starch. End-of-day starch content was approximately 1.65-fold more than controls while end-of-night starch content was approximately 1.98-fold more than controls (Figure 5A). We found that PGI plants did not linearly breakdown starch at night (*R*^2^ = 0.648), while empty vector plants did (*R*^2^ = 0.998) (Figure 5B). The Prob(F) value (likelihood that the regression parameters are zero) for the linear regression of empty vector plants was 7.64 × 10^–4^ while Prob(F) for PGI plants was 0.20 indicating linearity for the empty vector plants but not plants expressing retargeted AtPGI. Daytime synthesis of starch did not increase consistently throughout the day and was not further analyzed. Plants were grown in greenhouse conditions and the experiment took place on a cloudy day. Lights came on in the greenhouse late in the day (5:00 pm) and caused an increase in light compared to daytime intensity for the last 5 h of the photoperiod. Therefore, we focused on end of day, end of night, and night degradation values.

## Discussion

Plastidic PGI partitions carbon out of the Calvin-Benson cycle to starch synthesis, however, it is often assumed that it does not exhibit control over pathway flux. It is thought that ADP-glucose pyrophosphorylase (AGPase) exhibits most, if not all, of regulatory control over the starch synthesis pathway (Preiss and Sivak, 1998; Tiessen et al., 2002; Ballicora et al., 2004). Discussion of key enzymes and regulation of starch synthesis and computational models of starch synthesis often leave out early steps of the starch synthetic pathway, i.e., PGI and PGM, focusing instead on formation of ADP-glucose (ADPG) by AGPase and donation of glucose from ADPG to the growing starch chain (Preiss and Sivak, 1998; Tetlow et al., 2004; Sonnewald and Kossmann, 2014; Wu et al., 2014). However, the calculated flux-control coefficient for PGI can be 0.35 (Neuhaus and Stitt, 1990). Additionally, it has been shown that a loss of 50% of plastidic PGI reduces starch synthesis by 50% while loss of 64% of cytosolic PGI has a negligible effect on sucrose synthesis, reinforcing the rate-limiting role of the plastidic isoform (Kruckeberg et al., 1989). Due to its key role in controlling flux out of the Calvin-Benson cycle, a more robust understanding of PGI is necessary in order to understand the regulation of partitioning carbon out of the cycle.

### Three Levels of Regulation in Plastidic PGI

We have used recombinant enzymes to compare the plastidic isoform of PGI to the cytosolic. We have confirmed that the plastidic enzyme has a higher *K*_*m*_ for G6P than for F6P and that the cytosolic enzyme has similar *K*_*m*_’s for G6P and F6P. This makes it difficult for carbon to reenter the Calvin-Benson cycle as hexose phosphate. In addition to kinetic regulation, in the plastid the G6P/F6P ratio at equilibrium has been reported to be 3.70 at 25°C (Dyson and Noltmann, 1968). However, measurements from plastidic plant extracts show the ratio of G6P/F6P in the stroma to be close to 1 (Schnarrenberger and Oeser, 1974; Gerhardt et al., 1987; Sharkey and Vassey, 1989; Backhausen et al., 1997; Szecowka et al., 2013). In the cytosol, the G6P/F6P ratio is 2.4–4.7 (Gerhardt et al., 1987; Sharkey and Vassey, 1989; Szecowka et al., 2013). Kinetic isotope effects in starch, but not sucrose, also support the conclusion that plastidic PGI, but not cytosolic PGI, is unable to maintain equilibrium (Schleucher et al., 1999). Finally, our work suggests another mechanism of regulation. Using isolated chloroplasts to examine the *in vivo* kinetics of plastidic PGI, we have shown that the *K*_*m*_ of PGI can be dynamically regulated in the dark compared to the light. This may be due to post-translational modification or splice variants.

### Changes in Plastidic PGI Regulation Can Manipulate Starch Accumulation and Degradation

We found that targeting cytosolic PGI to the plastid affects starch synthesis and degradation. During the day, disruption of the normal expression of PGI in the plastid causes a ∼60% reduction in starch. This confirms the importance of PGI as a rate-determining step to partition carbon out of the Calvin-Benson cycle and also provides new avenues of investigation in engineering increased yields in crops.

We confirmed that the *K*_m_ for G6P of the plastidic enzyme is higher than that for F6P. In some cases a chloroplast envelope G6P transporter can be expressed, including in CAM plants and in response to a sudden increase in light (Weise et al., 2019). The properties of PGI will direct G6P entering the stroma toward starch and does not provide an easy route for G6P entry into the Calvin-Benson cycle. In mutants where GPT2 is expressed, G6P will enter the stroma but be unable to enter the Calvin-Benson cycle and will accumulate if starch synthesis cannot accommodate the influx of G6P. This can increase the activity of the plastidic G6PDH (Preiser et al., 2019) causing a G6P shunt that consumes ATP and, in some cases, requires cyclic electron flow, which compensates the ATP loss in the G6P shunt (Sharkey and Weise, 2016). This can explain the cyclic electron flow phenotype of many Calvin-Benson cycle mutants (Strand et al., 2017).

However, these results also demonstrate the need to consider other effects of manipulating flux out of the Calvin-Benson cycle. Targeting cytosolic PGI to chloroplasts, while increasing daytime starch accumulation, disrupted degradation of starch. Phosphorolytic starch breakdown results in the production of G1P and then G6P from starch and has been shown to be a significant contribution of carbon to the plastid (Weise et al., 2006). Mis-expressed PGI could increase degradation of starch by the phosphorolytic pathway by providing a pathway to enzymatic reactions that would not normally be available, in addition to the oxidative pentose phosphate pathway. This indicates that even at night PGI is an important regulatory point in starch metabolism and is a rate-limiting step that prevents early starch degradation through the night. The importance of nighttime activity of PGI was discussed relative to providing metabolites for the MEP pathway through the action of the oxidative pentose phosphate pathway, producing glyceraldehyde 3-phosphate (Bahaji et al., 2015). Many studies measure starch at end-of-day and end-of-night to assess effects on starch metabolism [see for example Scialdone et al. (2013)]. When manipulating partitioning from the Calvin-Benson cycle, future applications need to consider long-term consequences of early depletion of starch which may not be apparent in typical measurements.

### E4P Is a Strong Inhibitor of PGI

We previously assumed that PGA is a strong inhibitor of PGI [e.g., Sharkey and Weise (2016)] based on the report by Dietz (1985). Surprisingly, we did not observe this to be the case. Examination of data from Dietz (1985) shows that in PGA inhibition assays, 6PG was also present in the reaction mixture at 50 μM. The G6P/F6P disequilibrium in chloroplasts was proportional to PGA (Dietz, 1985) but PGA was not tested alone for its effect on PGI. We found that the *K*_*i*_ of plastidic PGI for 6PG with limiting F6P was 31 μM or with limiting G6P was 203 μM. Based on our findings, we propose that PGI is not inhibited by PGA, and the previously reported inhibition can be explained by presence of 6PG or E4P. *In vivo* plastidic concentrations of 6PG are not known, therefore, the extent of inhibition of PGI *in vivo* by 6PG cannot be currently determined.

Phosphoglucoisomerase is inhibited by μM concentrations of E4P (Grazi et al., 1960; Salas et al., 1964; Backhausen et al., 1997). E4P may be inhibitory to both isoforms of PGI because it is a competitive inhibitor and the active sites of both isoforms may be similar (Backhausen et al., 1997). Presumably there is no E4P in the cytosol since the cytosol lacks crucial enzymes in the non-oxidative branch of the pentose phosphate pathway (Schnarrenberger et al., 1995). Measurements and estimations of plastidic E4P concentrations *in vivo* show E4P to be ∼17–20 μM (Bassham and Krause, 1969; Heldt et al., 1977; Backhausen et al., 1997). This is well above the *K*_*i*_ of E4P for plastidic PGI. Backhausen et al. (1997) propose that this regulation is necessary in order to keep photosynthetic pool sizes stable during changes in light intensity.

In addition to stabilizing the Calvin-Benson cycle, we propose that inhibition of PGI by E4P can provide insight into the phenomenon of reverse sensitivity to CO_2_ and O_2_ of photosynthetic CO_2_ assimilation rate. This somewhat common behavior in leaves in high light and high CO_2_ can be explained in part by direct usage of glycine and serine from the photorespiratory pathway (Busch et al., 2018; Harley and Sharkey, 1991). However, in many cases the reverse sensitivity is greater than can be accounted for by this mechanism and is caused by a reduction in starch synthesis as reported by Sharkey and Vassey (1989). They proposed this was an effect of PGA inhibition of PGI, but because we did not find PGA to be inhibitory, we now suggest that the decrease in starch synthesis is due to an increase in E4P concentration (or possibly 6PG). On the other hand, Backhausen et al. (1997) found some inhibition of PGI by PGA but still they concluded that E4P was of particular interest in explaining control of PGI activity. It is possible that the degree of PGA inhibition of PGI is species-dependent but all available information clearly supports E4P as a powerful regulator of PGI.

Erythrose 4-phosphate inhibition of PGI may also explain some of the effects of sedoheptulose-1,7-bisphosphatase (SBPase) manipulation. SBPase is part of the Calvin-Benson cycle and converts sedoheptulose-1,7-bisphosphate to sedoheptulose 7-phosphate. It has been shown that overexpression of SBPase increases photosynthetic capacity and biomass (Miyagawa et al., 2001; Lefebvre et al., 2005; Rosenthal et al., 2011; Ogawa et al., 2015). Increased in SBPase activity would pull carbon forward in the Calvin-Benson cycle, reducing E4P levels. This would decrease PGI inhibition and partition more carbon toward starch synthesis. Conversely, reductions in SBPase decrease carbohydrates and total biomass (Harrison et al., 1998, 2001; Lawson et al., 2006). In this case, E4P may accumulate, decreasing PGI activity and the ability to partition carbon to starch. Decreased capability to synthesize starch has also been shown to decrease long-term triose phosphate usage capacity, reduce rubisco capacity, and limit RuBP regeneration (Yang et al., 2016).

## Conclusion

Redirection of carbon flux in chloroplasts to cause accumulation of a desired product is a common biotechnology goal. We conclude that PGI is an important regulatory enzyme in partitioning carbon out of the Calvin-Benson cycle. Previous analyses of the starch pathways have overlooked this key role. Additionally, we have re-examined previous knowledge of PGI inhibition and have found that it was mistakenly thought that PGI is inhibited by PGA and is instead inhibited by E4P.

## Data Availability Statement

The original contributions presented in the study are included in the article/Supplementary Material, further inquiries can be directed to the corresponding author.

## Author Contributions

All authors listed have made a substantial, direct and intellectual contribution to the work, and approved it for publication.

## Conflict of Interest

The authors declare that the research was conducted in the absence of any commercial or financial relationships that could be construed as a potential conflict of interest.

## Footnotes

1 https://www.genscript.com

2 http://www.sigmaaldrich.com

3 us.vwr.com

4 https://www.qiagen.com

5 http://www.thermofisher.com

6 https://www.icl-sf.com

7 http://www.cbs.dtu.dk/services/ChloroP/

8 https://www.retsch.com

9 https://moleculardevices.com
