# Supplementary material for: Phosphoglucoisomerase Is an Important Regulatory Enzyme in Partitioning Carbon out of the Calvin-Benson Cycle
Source: Front Plant Sci. 2020 Dec 10;11:580726. doi: 10.3389/fpls.2020.580726 (PMC7758399; doi:10.3389/fpls.2020.580726)
Supplement: Supplementary file 1 [file Data_Sheet_1.docx]

Supplementary Material

| Construct | Sequence |
| --- | --- |
| Cytosolic PGI | Gcgtcatcaaccgctttgatttgtgataccgaagcgtggaaggatttgaagggacatgtagaagatatt  aagaagactcatttgcgtgatttgatgagtgatgctaatagatgccagtccatgatgatggagtttgatgg  gttgctgttggattattctcgacagcgtgcaactgttgagacaatggacaagcttttgaacttggcaaagg  cttctcaattgacagagaagatcagccgcatgttcaatggggagcatattaacagtacagagaacagat  cagttcttcatgttgcgctccgtgctccaaaggatgcagttatcaaggctgatggaatgaatgtggttcca  gaagtgtggaacgttctagataagatcaaggaattttctgacaaaattcgctctggttcatgggttggagc  cactggcaaaccgctgaaagatgtcattgcgattggtattggtggtagcttcttaggtccactgtttgtcca  cacggctctccaaacagatcctgaagctctagagtctgctaaaggacgccagctgcgatttcttgcaaat  attgatcctgttgatgttgctagaaatatcagtggactaaatccagaaactactctagttgtggtggtctcga  aaacgtttacaacagctgaaacaatgcttaacgccagaacattgagggaatggataacagctgctcttgg  ggcttcagctgttgcaaaacatatggttgctgtcagcactaatcttgcgttagtagagaagtttggtattgac  ccgaacaatgcatttgcattttgggactgggttggtggaaggtacagtgtttgcagtgcggttggagtctta  cctttgtctctgcagtatggcttctccatggttgagaagtttttgaagggagcttcaagcattgatcagcattt  ccagtccacaccgttcgagaagaatatacctgtgcttttagggttgttgagtgtatggaatgtatcatttcttg  gatatcctgctagggccatcttaccttattcgcaagcccttgagaaatttgctccacacattcaacaggtta  gtatggagagtaatggaaagggagtctcaattgatggtctacctctcccgttcgagactggtgagattgat  tttggtgaacctggaacaaatggtcaacacagcttttaccaactcattcaccagggacgcgtaatcccttgt  gatttcattggcattgtgaagagtcagcaacctgtgtaccttaagggagaggtggtcagtaaccacgacg  agctcatgtcaaacttttttgcacagcctgatgctcttgcatatggaaaaactcctgaacagctgcagaaag  agaatgtttcagaaaatctcattccccataagacattctctggaaatcgaccttctcttagccttctacttcca  gaattgactgcttacaatgttggccagttgttggctatctatgaacacagagtagcagttcaaggctttgtg  tggggtatcaattcgtttgaccagtggggcgttgagctaggaaaagttctggctactcaggtcaggaaac  agcttcattcatcacgcactcaaggaaccgctcccgagggattcaattacagtaccaccacacttttgaaa  cgatatctggagacaagttccgagccccagatg |
| YFP | Gtgagcaagggcgaggagctgttcaccggggtggtgcccatcctggtcgagctggacggcgacgtaaa  Cggccacaagttcagcgtgtccggcgagggcgagggcgacgccacctacggcaagctgaccctgaa  Gttcatctgcaccaccggcaagctgcccgtgccctggcccaccctcgtgaccaccttcggctacggcctg  Aagtgcttcgcccgctaccccgaccacatgaagcagcacgacttcttcaagtccgccatgcccgaaggct  Acgtccaggagcgcaccatcttcttcaaggacgacggcaactacaagacccgcgccgaggtgaagttcg  Agggcgacaccctggtgaaccgcatcgagctgaagggcatcgacttcaaggaggacggcaacatcctgg  Ggcacaagctggagtacaactacaacagccacaacgtctatatcatggccgacaagcagaagaacggcat  Caaggtgaacttcaagatccgccacaacatcgaggacggcagcgtgcagctcgccgaccactaccagca  Gaacacccccatcggcgacggccccgtgctgctgcccgacaaccactacctgagctaccagtccgccctg  Agcaaagaccccaacgagaagcgcgatcacatggtcctgctggagttcgtgaccgccgccgggatcactc  tcggcatggacgagctctacaagtga |
| Transit peptide | Atggcctctctctcaggcctatactcttcttctccatctctcaaacctgccaaaaaccattcctttaaagcattgc  cggcgcaatctagagattccttctctttcccacatacctccaaacccaccaatctaccgttgactctc |

Supplementary Table 1. Transient expression construct sequences

Supplementary Figure 1. SDS-PAGE of purified PGI proteins, stained with Coomassie blue.

Supplementary Figure 2. Effect of F6P and G6P on plastidic (A) and cytosolic (B) AtPGI specific activity.


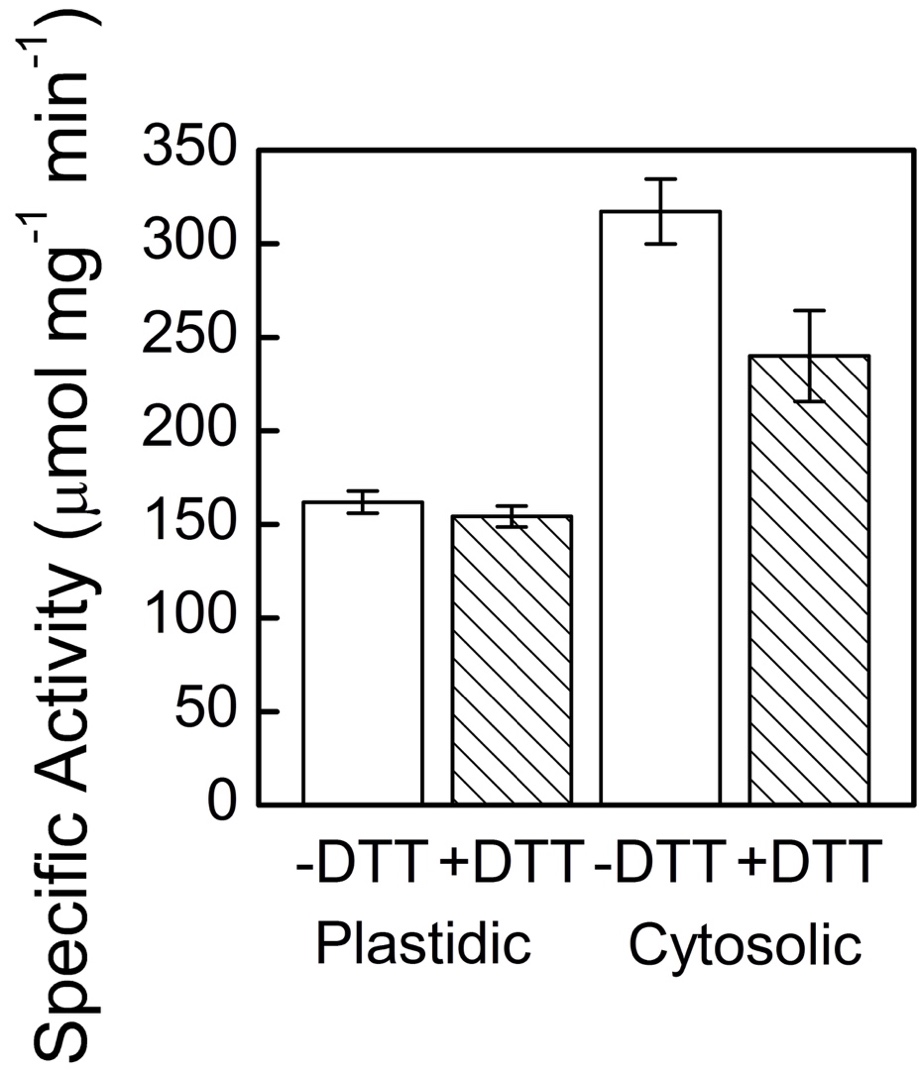


Supplementary Figure 3. Specific activity of plastidic and cytosolic AtPGI with and without 10 mM DTT. The activities were not statistically different.


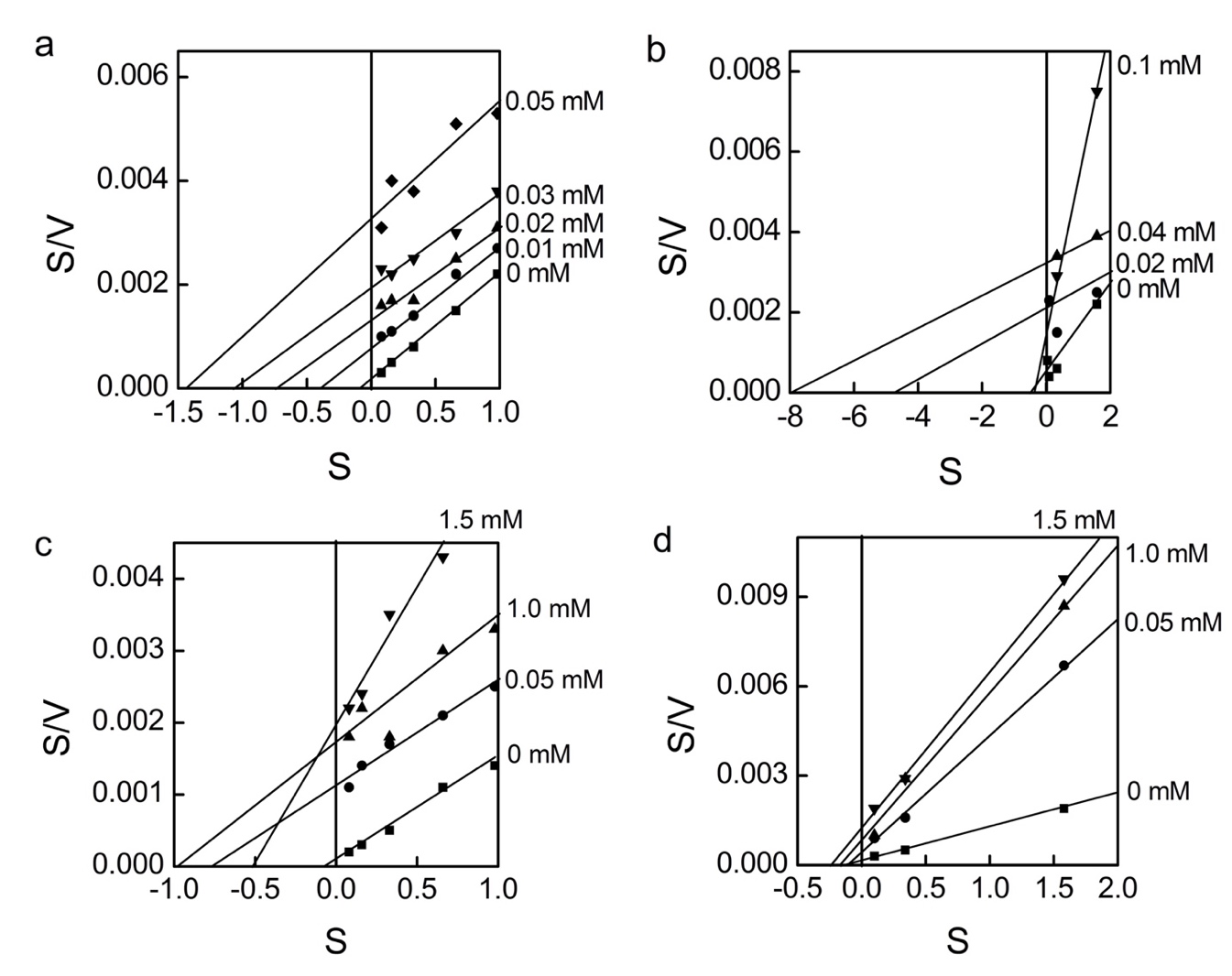


Supplementary Figure 4. Hanes-Woolf plots of E4P (a, b) and 6PG (c, d) inhibition of plastidic AtPGI with G6P as a substrate. Lines represent linear regression by least squared error.


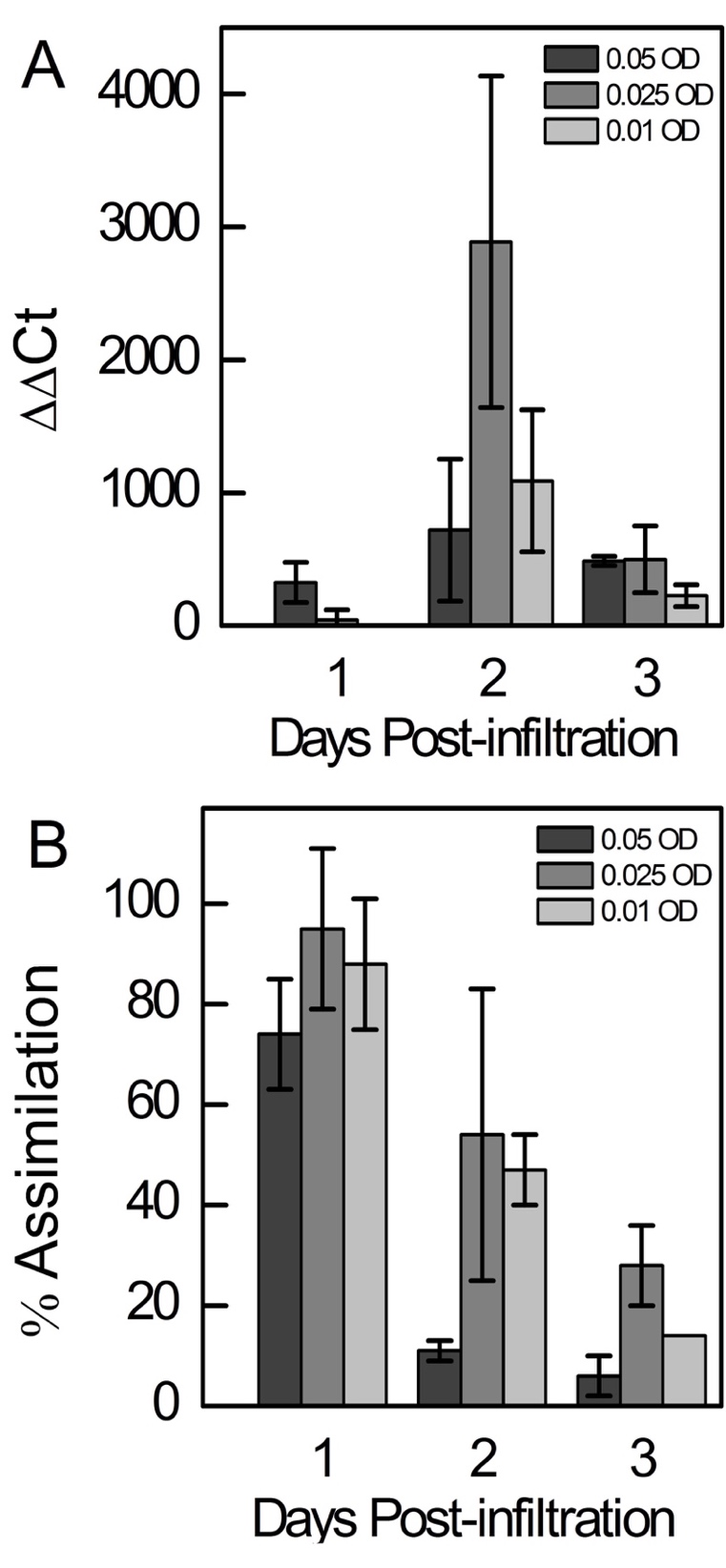
Supplementary Figure 5. qPCR (A) and rates of assimilation (B) for *N. tabacum* infiltrated with mislocalized pPGI. All values are relative to pre-infiltration samples from the same plant.
